# A patient with X-linked adrenoleukodystrophy presenting with central precocious puberty: a case report

**DOI:** 10.1007/s12020-023-03562-w

**Published:** 2023-10-16

**Authors:** Ting Ting Zhu, Jin Wu, Xiao Mei Sun

**Affiliations:** 1 Department of Pediatrics, West China Second University Hospital, Sichuan University, Chengdu, China; 2 Key Laboratory of Obstetric & Gynecologic and Pediatric Diseases and Birth Defects of Ministry of Education, Sichuan University, Chengdu, Sichuan China

**Keywords:** X-ALD, ALDP, VLCFAs, CPP

## Abstract

X-linked adrenoleukodystrophy (X-ALD) is a peroxisomal disorder caused by the variations in the ATP-binding cassette sub-family D member 1 (ABCD1) gene. This study is the first to report central precocious puberty (CPP) in individuals with X-ALD. A 6-year-old boy exhibited mucocutaneous pigmentation, increased plasma adrenocorticotropic hormone levels, and elevated very long-chain fatty acids (VLCFA). We identified a variant, c.1826A>G (p. Glu609Gly), in exon 8 of the ABCD1 gene in the proband. Additionally, he displayed rapid growth, testicular volume of 5–6 mL, the onset of pubic hair, and pubertal levels of luteinizing hormone (LH), all meeting the diagnostic criteria for CPP.

## Introduction

X-linked adrenoleukodystrophy (ALD) is a metabolic disorder characterized by impaired peroxisomal beta-oxidation of very long-chain fatty acids (VLCFA; ≥ C22). ALD is caused by mutations in the ATP-binding cassette sub-family D member 1 (ABCD1) gene, responsible for encoding the peroxisomal transmembrane protein (also known as ALDP), which facilitates VLCFA transport into the peroxisome for β-oxidation. An ALDP malfunction results in VLCFA accumulation in all tissues, with the most significant increase observed in the brain, spinal cord, adrenal cortex, and Leydig cells of the testes [1–3].

Central precocious puberty (CPP) is characterized by the appearance of any sign of secondary sexual maturation before the age of 8 years in girls and 9 years in boys, resulting from the activation of the hypothalamic-pituitary-gonadal (HPG) axis [4]. To date, there have been no reports of CPP in ALD patients. In this report, we present a case of ALD and CPP in a male patient. We have identified a variant, c.1826A>G (p. Glu609Gly), in exon 8 of the ABCD1 gene of the proband.

## Case report

A 6 year and 8-month-old boy was referred to our hospital because of mucocutaneous pigmentation that had commenced around the age of 5. At 3 years old, he had experienced a brief coma after vomiting and diarrhea, which had resulted in his hospitalization (specific details are unavailable). No issues related to vision, hearing, mobility, or cognitive function were observed in him. No abnormalities were detected during the neurological examination. Notably, his uncle had succumbed to diarrhea at the age of 1, and paralysis had been experienced by his maternal granduncle around the age of 40.

His measurements were as follows: height of 124.5 cm (Z score, 0.39), weight of 23.5 kg (Z score, 0.04), and a body mass index of 15.16 kg/m² (Z score, −0.21). An eight o’clock cortisol measurement showed a level of 61.7 nmol/L (adrenal insufficiency threshold: <83 nmol/L; adrenal insufficiency was ruled out with a level of > 414.6 nmol/L). Plasma adrenocorticotropic hormone (ACTH) level exceeded 2000.0 pg/mL (reference range, [< 46 pg/mL]). The ACTH stimulation test demonstrated that cortisol levels at 0, 30, and 60 min were measured at 69.2, 61.7, and 68.6 nmol/L, respectively (reference range, [> 500 nmol/L is considered normal]), and the17-hydroxyprogesterone levels at 0, 30, and 60 min were measured at 0.1, 0.5, and 1.2 nmol/L, respectively. The plasma very long-chain fatty acids (VLCFA) assay (Table 1) revealed the following results: C26:0, 0.398 μmol/L (normal range, 0.008–0.085 μmol/L); C24:0, 0.319 μmol/L (normal range, 0.015–0.172 μmol/L); C24:0/C22:0 ratio, 1.195 (normal range, 0.124–1.087), and C26:0/C22:0 ratio, 5.307 (normal range, 0.183–2.316), indicating elevated plasma VLCFA levels. Magnetic resonance imaging of the brain, hypophysis, and adrenal gland revealed normal findings.

**Table 1:** a. Clinical characteristics of the reported patient who had central precocious puberty at the diagnosis of ALD. b. Very Long Chain Fatty Acid Level (μmol/L) in the Patient and Reference Values

Characteristics	Value		Characteristics		Value
**a**					
Age at diagnosis, years	6		Serum ACTH, pg/ml		> 2000
Height, cm (SDS)	124.5 (0.39)		Serum cortisol at eight o’clock, nmol/L		61.7
Weight, kg (SDS)	23.5 (0.04)		Serum LH, IU/L		0.4
Body mass index, kg/m2 (SDS)	15.16 (−0.21)		Serum FSH, IU/L		1.1
Bone age, years	8		Serum 25-hydroxyvitamin D, ng/mL		30.4
Testicular volume, mL	5–6		Serum calcium, mmol/L		2.34
**b**					
C20:0-LPC		0.267		0.031–0.404	
C22:0-LPC		0.075		0.008–0.119	
C24:0-LPC		0.319↑		0.015–0.172	
C26:0-LPC		0.398↑		0.008–0.085	
C24/C20		1.195↑		0.124–1.807	
C26/C20		1.491↑		0.066–0.716	
C24/C22		4.253↑		0.481–3.172	
C26/C22		5.307↑		0.183–2.316	

↑ indicate the result higher than reference value.

In addition to the clinical features mentioned above, a rapid growth velocity of 8 cm/year (SDS > 1) was observed. Pubertal examination revealed a testicular volume of 5–6 mL measured by Prader orchidometer and a penile length of 6 cm with sparse pubic hair. Random luteinizing hormone (LH), follicle-stimulating hormone (FSH), and testosterone levels were measured at 0.4 IU/L, 1.1 IU/L, and 0.77 ng/mL (reference range, [< 0.07 ng/mL is considered prepubertal concentrations]), respectively. The bone age, evaluated by TW2 score systems, was determined to be 8 years and 4 months. Further GnRH stimulation test reported that the peak level of LH was measured at 12.5 IU/L and FSH 3.1 IU/L. The boy was considered to have CPP due to the accelerated growth, enlargement of the testes, appearance of pubic hair, advanced bone age, and elevated LH before the age of 9.

Whole-exome sequencing of the peripheral blood of the proband revealed a variant, c.1826A>G (p. Glu609Gly), in exon 8 of the ABCD1 gene. The inheritance mode was determined to be X-linked recessive, with the proband being hemizygous, and the mother being heterozygous. The pathogenicity of the genetic variation, based on the American College of Medical Genetics criteria, was identified as uncertain, with support from PM5 + PM2_Supporting+PP3 (PM, pathogenic moderate; PP, pathogenic supporting). The same variant had been identified as pathogenic in four ALD cases [5–7] (https://www.ncbi.nlm.nih.gov/clinvar/variation/528345/).

The patient underwent glucocorticoid replacement therapy, taking hydrocortisone orally at a dose of 15 mg/m²/day, administered in three daily doses. Plasma cortisol level was measured at 225 nmol/L two hours after the ingestion of hydrocortisone (hydrocortisone has a half-life of 100 min). The patient responded well to hydrocortisone treatment, with normal blood pressure, blood glucose levels, and blood electrolyte levels. The mucocutaneous pigmentation went into remission, and there were no notable neurological disorders observed.

Although diagnosed with CPP, the patient did not receive gonadotropin-releasing hormone agonist (*GnRHa*) treatment immediately. Observation for 6 months will help to decide whether the puberty is progressing rapidly [8]. After 6 months of observation, the growth velocity was 6 cm/year (SDS = 1), testicular volume was 6 mL, and the bone age advanced by 5 months over six months. Random LH and testosterone level were measured at 0.5 IU/L and 0.87 ng/mL, respectively. These manifestations indicated a slow progression of puberty which does not require GnRHa treatment. Now the patient is in another 6 months of follow-up observation.

This study received approval from the Ethics Committee of West China Second Hospital, Sichuan University. Written informed consent was obtained from the patient and the patient’s parents.

## Discussion

X-ALD displays a broad clinical spectrum, with three primary phenotypes: childhood cerebral form, adrenomyeloneuropathy (AMN), and “Addison disease only.” In males, the onset of “Addison disease only” can occur anytime between 2 years of age and adulthood, but it typically emerges before 7.5 years, with no apparent neurological abnormalities. Symptoms include unexplained vomiting, weakness, and coma due to cortisol insufficiency, along with hyperpigmentation due to increased ACTH [9]. In our case, the boy experienced vomiting and coma symptoms but received a delayed diagnosis until mucocutaneous pigmentation appeared and plasma VLCFA levels were tested, ultimately confirming X-ALD clinically. It is worth noting that X-ALD may be underdiagnosed in boys with adrenal insufficiency, as one study found that 83% of boys with unexplained adrenal insufficiency had X-ALD [10, 11]. Plasma C26:0/C22:0 and C24:0/C22:0 ratios are the diagnostic hallmarks of ALD [12]. Elevated plasma VLCFA levels are observed in nearly all male X-ALD patients, regardless of clinical severity, and in 85% of female carriers [13]. Therefore, testing plasma VLCFA levels provides an opportunity for screening patients with X-ALD.

To date, 1405 different variants of the ABCD1 gene have been documented in the ALD Mutation Database [14]. However, no clear genotype-phenotype correlations have been identified. In our study, whole-exome sequencing analysis revealed a variant, c.1826A>G (p. Glu609Gly), in exon 8 of the ABCD1 gene in the proband. Notably, the patient’s uncle and maternal granduncle had suspected X-ALD but were not genetically tested, as they passed away years ago.

No prior studies have reported CPP in individuals with X-ALD. The underlying mechanism of the activation of the HPG axis and subsequent entry timely into puberty is complicated and has not yet been fully clarified. Specific genetic mutations, central nervous system diseases, some rare syndromes, sex steroids exposure, and environmental factors are the major causes of CPP [15]. Not all patients with CPP require GnRHa treatment. GnRHa treating CPP primarily include patients who are young and also demonstrate a rapid progression of pubertal changes [8]. In our study, the patient was followed up for six months, and a slow progression puberty was observed, so he was not given GnRHa. However, continued observation and follow-up are needed.

The connection between X-ALD and precocious puberty has been perplexing. To the best of our knowledge, the disturbances of VLCFA can result in hypogonadism [16]. Previous studies have detected testicular dysfunction in adult males with X-ALD, characterized by elevated FSH and LH levels and lower testosterone levels, whereas no signs of testicular insufficiency were observed in prepubertal ALD patients [2, 16, 17]. In contrast to hypogonadism, our patient developed CPP, marking the first reported case of CPP in ALD. Höftberger et al. investigated the expression of ABCD1 in a broad range of human tissues and found ALDP was also strongly expressed in the hypothalamus, in addition to the subcortical and cerebellar white matter in the brain [18]. This may explain disruptions in the HPG axis in X-ALD patients. We hypothesize that the CPP in this X-ALD boy might progress to partial testicular dysfunction in adulthood, necessitating further follow-up.

In summary, we present a case of X-ALD in a boy with CPP, accompanied by the identification of a ABCD1 gene variant. VLCFA analysis plays a pivotal role in ALD diagnosis. It is imperative to assess and monitor sexual development in ALD patients.

## Data Availability

All data supporting the findings of this article are included in the manuscript.
